# Development and Validation of Pharmacology Concept Inventory for Concept‐Based Learning: Leveraging Theory, Expert Insights, and Student Perspectives

**DOI:** 10.1002/prp2.70237

**Published:** 2026-03-22

**Authors:** Adeladlew K. Netere, Tony Hughes, Anna‐Marie Babey, Clare Guilding, Carolina Restini, Martin Hawes, John P. Kelly, Elvan Djouma, Jennifer Koenig, Jacqueline E. McLaughlin, Olusola Olafuyi, Lynette B. Fernandes, Janet Mifsud, Graeme J. Sills, Anneke H. van Houwelingen, Steven J. Tucker, Willmann Liang, Patrik Aronsson, Farhan Ahmad Khan, Tina Hinton, Mark Hernandez, Lindsay Cormier, Roisin Kelly‐Laubscher, Fabiana A. Caetano Crowley, Marina Junqueira Santiago, Margaret Cunningham, Jennelle Durnett Richardson, Kelly Karpa, Paul J. White

**Affiliations:** ^1^ Faculty of Pharmacy and Pharmaceutical Sciences Monash University Parkville Victoria Australia; ^2^ School of Science & Technology University of New England Armidale New South Wales Australia; ^3^ Faculty of Medical Science, School of Medicine Newcastle University Newcastle upon Tyne UK; ^4^ Department of Pharmacology and Toxicology Michigan State University East Lansing Michigan USA; ^5^ Department of Veterinary Clinical Sciences, School of Veterinary Medicine University of Surrey Guildford UK; ^6^ Discipline of Pharmacology and Therapeutics, School of Pharmacy and Medical Sciences University of Galway Galway Ireland; ^7^ Department of Microbiology, Anatomy, Physiology and Pharmacology, School of Agriculture, Biomedicine and Environment La Trobe University Melbourne Victoria Australia; ^8^ School of Medicine University of Nottingham Nottingham UK; ^9^ Division of Practice Advancement and Clinical Education, UNC Eshelman School of Pharmacy University of North Carolina Chapel Hill North Carolina USA; ^10^ Division of Physiology, Pharmacology and Neuroscience, School of Life Sciences University of Nottingham Nottingham UK; ^11^ School of Biomedical Sciences The University of Western Australia Perth Western Australia Australia; ^12^ Department of Clinical Pharmacology and Therapeutics University of Malta Msida Malta; ^13^ School of Cardiovascular & Metabolic Health University of Glasgow Glasgow UK; ^14^ Department of Pharmaceutical Sciences, Faculty of Science Utrecht University Utrecht the Netherlands; ^15^ School of Medicine, Medical Science and Nutrition University of Aberdeen Aberdeen UK; ^16^ Department of Pharmacology and Pharmacy, LKS Faculty of Medicine The University of Hong Kong Hong Kong SAR Hong Kong; ^17^ Department of Pharmacology, Institute of Neuroscience and Physiology, Sahlgrenska Academy University of Gothenburg Gothenburg Sweden; ^18^ Department of Pharmacology, Jawaharlal Nehru Medical College Aligarh Muslim University Aligarh India; ^19^ Faculty of Medicine and Health, Sydney Pharmacy School The University of Sydney Sydney New South Wales Australia; ^20^ Department of Medical Education, Quillen College of Medicine East Tennessee State University Johnson City Tennessee USA; ^21^ Department of Pharmacology and Nutritional Sciences, College of Medicine University of Kentucky Lexington Kentucky USA; ^22^ Department of Pharmacology and Therapeutics, School of Medicine, College of Medicine and Health University College Cork Cork Ireland; ^23^ Department of Physiology & Pharmacology, Schulich School of Medicine & Dentistry Western University London Ontario Canada; ^24^ Macquarie Medical School Macquarie University Sydney New South Wales Australia; ^25^ Strathclyde Institute of Pharmacy and Biomedical Sciences (SIPBS) University of Strathclyde Glasgow UK; ^26^ Department of Biochemistry, Molecular Biology, and Pharmacology Indiana University School of Medicine Indianapolis Indiana USA; ^27^ Department of Medical Education, East Tennessee State University Quillen College of Medicine Johnson City Tennessee USA

**Keywords:** core concepts of pharmacology, development, pharmacology concept inventory, pilot study, validation

## Abstract

Misconceptions in pharmacology can undermine learning and compromise both clinical and scientific reasoning, yet few validated tools exist to identify them. Consequently, we developed and validated the Pharmacology Concept Inventory (PCI), which can be used to identify misconceptions, assess learning gains, and evaluate teaching effectiveness. This PCI was designed based on the IUPHAR‐Education Section (IUPHAR‐Ed) Core Concepts of Pharmacology Project, addressing eight core concepts: drug efficacy, drug‐target interaction, steady‐state concentration, structure–activity relationship, drug tolerance, drug bioavailability, volume of distribution, and drug clearance. A triangulated design strategy integrated theoretical frameworks, expert review, and student perspectives. Experts examined quality, content validity, and cognitive alignment. The pilot PCI was then administered to a student cohort to evaluate its psychometric properties, providing preliminary evidence for further refinement. Item‐level content validity indices ranged from 0.67 to 1.00, with a scale‐level average of 0.93. Seventy students completed the pilot survey, leading to the exclusion of items with low discrimination and reliability. Items on drug‐target interaction were removed due to consistently poor performance. The final PCI included 26 items covering seven concepts, with strong discrimination indices (0.36–0.75) and difficulty indices (0.26–0.71). Internal consistency was high (Cronbach's alpha = 0.91), and concept‐level reliability ranged from 0.64 to 0.85. The PCI provides strong evidence for identifying misconceptions and assessing learning outcomes through a pre–post‐test approach. Although the PCI currently addresses only a subset of concepts, continued refinements informed by surveys and interviews will enhance its utility and expand its scope for concept‐based learning and curriculum evaluation.

**Abbreviations**
I‐CVI: Item Content validity index
IUPHAR‐Ed CCP: International Society for Basic and Clinical Pharmacology Education Section Core Concepts of Pharmacology
PCI: Pharmacology concept inventory
PD: pharmacodynamic(s)
PK: pharmacokinetic(s)
S‐CVI: scale‐level content validity index

## 1 Introduction

Concept inventories are research‐based, multiple‐choice tools designed to evaluate learners' understanding of fundamental concepts and to identify persistent misconceptions [1]. Test items include one accurate response and several student‐derived distractors elicited from open‐ended responses, interviews, prior research, and expert review, such that incorrect choices reflect authentic alternative conceptions [2, 3, 4]. In concept inventory research and practice, these distractors provide teachers with diagnostic insights that supports targeted instruction and classroom implementation. In addition, concept inventories efficiently reveal the prevalence and patterns of misconceptions across groups [5, 6, 7].

Concept inventory tools are systematic, theory‐based, student‐centered instruments used to assess the frequency and persistence of misconceptions and to compare conceptual outcomes across groups or instructional methods. Norris [6] noted that concept inventories are “commonly used by researchers and educators to gauge the prevalence of student misconceptions in science,” while Klymkowsky and the team [8] emphasized that “a well‐designed concept test can indeed reveal the presence and/or absence of conceptual confusion.” Their development follows principles from cognitive psychology and educational measurement, ensuring construct validity [9, 10]. Cognitive psychology underscores the limited capacity of working memory, highlighting the importance of concise, focused items to avoid overload. Item‐writing guidelines warn against unnecessary complexity or irrelevant difficulty, which can distort measurement [2, 11]. Educational measurement principles, such as item response theory, model the interaction between student ability and item difficulty, thereby enhancing reliability and fairness [12]. Sadler [4] argued that integrating psychometric models with distractor‐based assessments links qualitative insights into student thinking with quantitative measures of enduring alternative conceptions, providing evidence to target pedagogical strategies [8].

The adoption of concept inventories has become widespread across disciplines such as physics, chemistry, biology, astronomy, physiology, and oceanography [3, 13], with many tools developed over the past decade. This trend is linked to their perceived usefulness and effectiveness in enhancing educational outcomes. Concept inventories assist educators pinpoint areas in which students' misconceptions are clustered, enable implementation of targeted instruction and interventions [14], and support benchmarking across courses and institutions, curriculum development, and discipline‐based education research [13, 15, 16]. A scoping review reported that 80% of concept inventory tools focused on measuring conceptual understanding, 48% identified the prevalence of misconceptions, 40% assessed both, 26% measured learning gains, and 22% investigated instructional effectiveness [3]. Concept inventories are also used to compare cohorts and pedagogies when validity supports such comparisons [14, 17]. While learning gains may suggest trends in teaching effectiveness, they should be interpreted cautiously and triangulated with other evidence [8, 14].

Experiences from physics have demonstrated how concept inventories support learning assessment and continuous improvement [18]. Through pre–and post‐tests, such tools reveal persistent misconceptions, track conceptual understanding, and guide the development of targeted instructional interventions [14, 19]. A large body of evidence has demonstrated that students engaged in active‐learning approaches performed better on examinations and achieved higher inventory scores in science, technology, engineering and mathematics (STEM) fields [20]. Collectively, these applications assist in refining teaching methods, aligning curricula with learning needs, and enabling valid comparisons across instructional approaches, provided that methods are valid and reliable, items are consistently interpreted by cohorts, and appropriate statistics are used [17].

Despite the widespread use of concept inventories across STEM fields [3], pharmacology lacks such an instrument for assessing understanding of fundamental concepts. Developing one would provide an evidence‐based way to examine how students' responses align with documented misconceptions, support concept‐driven learning, and help in designing and evaluating instructional strategies. This gap is especially significant given the scope of pharmacology as a discipline. Pharmacology is the science of drug‐organism interactions, vital for therapeutic optimization, safe prescribing, alleviating clinical symptoms, improving prognoses, extending life, and preventing disease. Due to its clinical importance, a firm grasp of core pharmacology concepts is essential for students in healthcare professions, biomedical sciences, pharmaceutical sciences, and basic sciences. As a result, pharmacology remains a key discipline taught across undergraduate, graduate, and postgraduate levels, employing various teaching methods such as theoretical lessons, problem‐based learning, and laboratory activities [21, 22].

Unfortunately, it has been shown that healthcare professionals often have marked gaps in their understanding of pharmacology [23, 24]. A focus group study with Australian clinical nurses revealed that they often find it challenging to understand and apply basic pharmacology concepts in practice [25]. Recent studies [26, 27] by the International Society for Basic and Clinical Pharmacology Education Section (IUPHAR‐Ed) Core Concepts of Pharmacology Project investigated students' understanding of selected pharmacokinetics (PK) and pharmacodynamics (PD) core concepts across 12 universities worldwide, involving students in medical, pharmacy, veterinary, pharmaceutical science, and related programs. This study did not examine all core concepts, focusing instead on eight Core Concepts of Pharmacology, including drug clearance, drug bioavailability, steady‐state concentration, volume of distribution, drug efficacy, drug tolerance, drug–target interactions, and structure–activity relationships. The results highlighted the need for systematic, theory‐based tools grounded in a student‐centered learning environment. As a result, developing a Pharmacology Concept Inventory (PCI) could provide a systematic way to identify persistent misconceptions and conceptual confusions, support concept‐based learning, and guide the design and evaluation of teaching strategies in pharmacology education.

### 1.1 Core Concepts of Pharmacology Establishment

The development of a PCI was grounded in the IUPHAR‐Ed Core Concepts of Pharmacology project, which defined the fundamental concepts essential for pharmacology research and therapy [28, 29]. The project identified 24 core concepts and refined them into 103 sub‐concepts, providing a validated framework that ensures the PCI is aligned with global consensus key concepts in pharmacology.

Guided by the Core Concepts of Pharmacology framework, the present study aimed to develop and validate a pilot PCI as a diagnostic tool to assess students' understandings and misconceptions across a subset of these core concepts. Additionally, the study aimed to establish criterion‐based content validity, conduct item analysis, and evaluate internal consistency, thereby strengthening the tool's psychometric foundations and facilitating iterative refinement. Ultimately, the PCI is intended to serve as an evidence‐based resource to support the development of a concept‐based pharmacology curriculum. This effort could further enhance interactive learning methods, promote a cohesive evaluation approach, and address knowledge gaps within the curriculum.

## 2 Methods

### 2.1 Ethics Statement

Ethics approval was obtained from the Monash University Human Research Ethics Committee (MUHREC, approval protocol ID: 45836).

### 2.2 Pharmacology Concept Inventory (PCI) Design Framework

**FIGURE 1:**
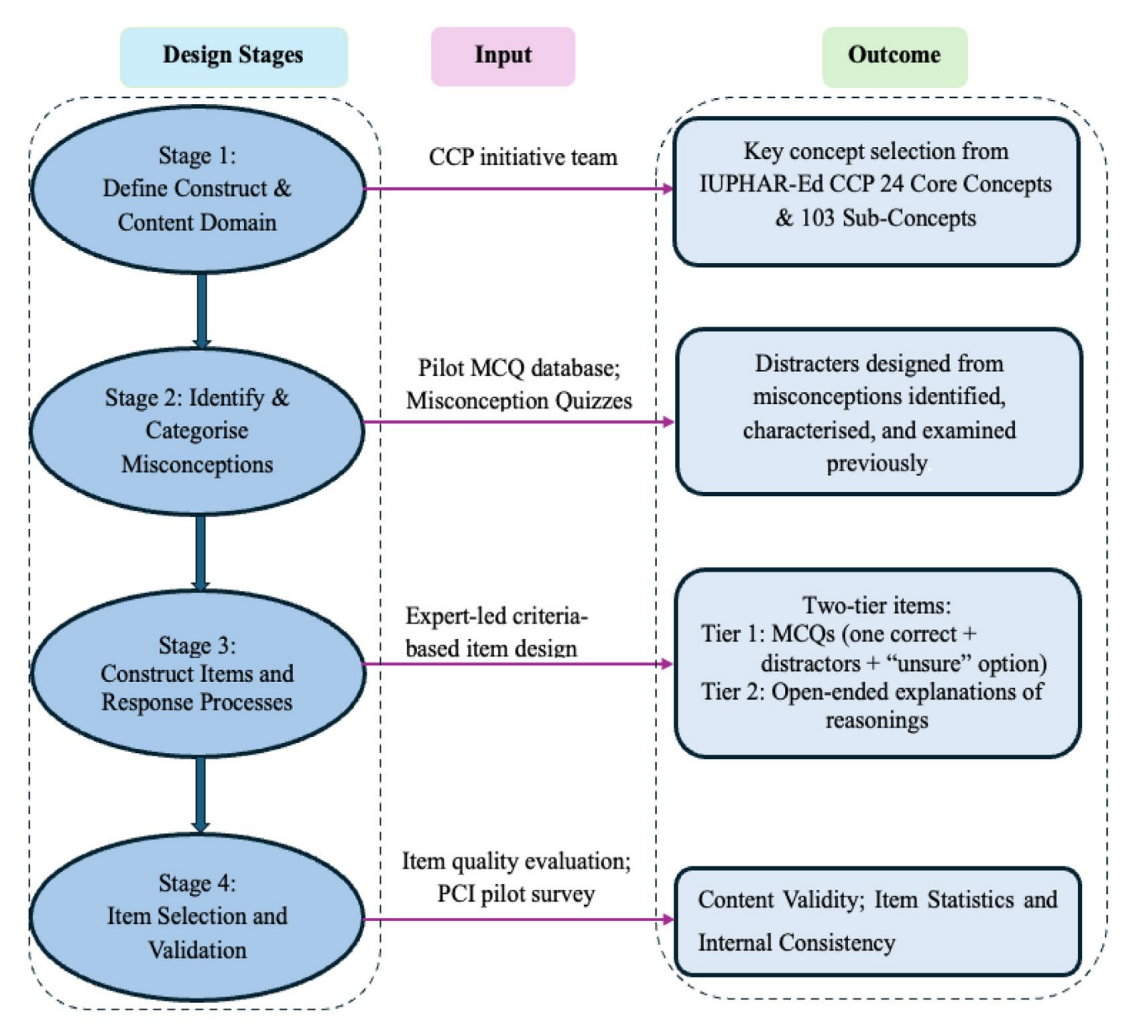
Design framework for the Pharmacology Concept Inventory (PCI) Tool. IUPHAR‐Ed CCP, International Society for Basic and Clinical Pharmacology Education Section Core Concepts of Pharmacology. The framework illustrates the design process used to develop the PCI. Stages 1 and 2 drew on previously published IUPHAR‐Ed CCP initiative projects. Stage 1 involved selecting core and sub‐concepts of pharmacology for item development [28, 29]; Stage 2 examined documented misconceptions to design plausible item distractors [26, 27, 30].

The development and validation of the PCI were guided by learning theories, expert insights, and student perspectives. The pilot design process followed four key stages (Figure 1).

#### 2.2.1 Stage 1: Concepts Selected, and Content Domain Determined

The construct was defined, the content domain delineated, and eight key concepts selected from the IUPHAR‐Ed Core Concepts of Pharmacology Project [28, 29]. This project employed an experimental and iterative process of explanation and refinement, incorporating surveys, workshops, and expert review to establish globally endorsed core concepts. This process provided an evidence‐based foundation for PCI item development grounded in practical educational experience.

#### 2.2.2 Stage 2: Misconceptions Examined, and Distractors Designed

Student‐driven misconceptions were examined to inform distractor construction. Sources included international student‐informed misconception quizzes [26, 27] and an educator‐based multiple‐choice question (MCQ) pilot database [30], which were used to design distractors for the pilot PCI tool development. This dual‐source approach ensured that the PCI reflected both expert insights and student perspectives. The present study focused on the first eight core concepts, including drug clearance, drug bioavailability, steady‐state concentration, volume of distribution, drug efficacy, drug tolerance, drug–target interactions, and structure–activity relationships identified from the pilot study (Figure S1) [26, 27].

#### 2.2.3 Stage 3: Item Constructed, and Response Formatted

Items were designed in a two‐tier format to improve diagnostic accuracy. The first tier included one correct answer and distractors reflecting common misconceptions, along with an additional “*unsure*” option to minimize random guessing and prevent forced‐choice responses. The second tier asked students to give an open‐ended justification (“Explain why you chose this option”), enabling a detailed assessment of their reasoning and understanding. An example of the item design and its iterative refinement process is shown in the supporting material (Figure S2). Using scale development theory [31, 32, 33, 34], the initial item pool was created by experts who refined existing items from the pilot MCQ database [30] and developed new items based on findings from misconception quizzes [26, 27].

#### 2.2.4 Stage 4: Pharmacology Concept Inventory (PCI) Item Quality Evaluation and Pilot Survey

Items were evaluated through a two‐step process. First, experts employed a criterion‐based quality assessment framework to review clarity, content and cognitive alignment [30]. Second, a student pilot test was conducted to generate empirical evidence on item statistics, internal consistency, and overall performance. Together, these complementary evaluations provided robust psychometric evidence to guide the refinement of the PCI.

### 2.3 Expert Team Composition

Twenty‐eight international pharmacology educators were invited via email from the Core Concepts of Pharmacology Project to participate in item development and overall quality assessment. These experts were selected from four continents and ten countries (Australia, Canada, Hong Kong, India, Ireland, Malta, Sweden, the Netherlands, the United Kingdom, and the United States) based on their expertise in pharmacology, extensive teaching experience in foundational and systems‐based pharmacology, and prior item development experience. The panel included 16 women and 11 men, with one expert opting not to disclose. The majority (*n* = 25) hold PhDs, while the remaining two have Master's degrees. Their expertise spans fields including pharmacology, pharmacy, veterinary medicine, medicinal chemistry, natural sciences, endocrinology, psychiatry, and education. Their academic specializations encompass pharmacology, pharmacotherapeutics, pharmacokinetics, and medicinal chemistry, and they teach across disciplines such as medicine, pharmacy, veterinary medicine, dentistry, nursing, psychology, biomedical sciences, and allied health. Their teaching responsibilities extend from undergraduate to postgraduate levels across programs like pharmacy, health sciences, biomedical sciences, physician assistant, dental, medical, life sciences, paramedicine, veterinary medicine, and podiatry. Nearly half (*n* = 13) have over 20 years of teaching experience, and about a third (*n* = 10) have at least 11 years, in which their class sizes ranging from 50 to over 250 students (Table S1).

### 2.4 Quality Evaluation and Content Validity

Content validity is defined as “the degree to which elements of an instrument are relevant to and representative of the targeted construct for a particular assessment purpose” [35] and was established through expert review by pharmacology educators. This process ensured that each item in a PCI demonstrated clarity, accuracy, and alignment with the intended core concept. The review focused on determining the extent to which each item reflected the content domain that was comprehensible to test‐takers and collectively represented the full scope of the core concept [35, 36].

In line with the quality analysis framework proposed by Netere and the team [30], the PCI pilot underwent pre‐administration quality evaluation and content validation across three dimensions: alignment with the Core Concepts of Pharmacology framework [28, 29], appropraite cognitive level based on Bloom's taxonomy [37, 38] and compliance with established item‐writing guidelines [30]. Items' conceptual coverage was rated from inadequate (leveled as 0) to encompassing the main idea (3) of the core concepts assessed. Cognitive demand was classified according to Bloom's taxonomy, distinguishing between lower‐order (knowledge, comprehension) and higher‐order (application, analysis, synthesis, evaluation) cognitive classes. Item‐writing quality was graded as high (met all item‐writing guidelines), good (met 20–22), or fair (met 18–19), ensuring clarity, validity, and reliability [30].

Following this evaluation, items were reviewed for alignment with the eight core concepts, leading to the removal of items that were conceptually overlapping or misaligned. Content validity was measured using the Content Validity Index (CVI) at both the item level (I‐CVI) and scale level, with the average method (S‐CVI/Ave), in accordance with established practices [39]. The S‐CVI/Ave reflects the extent to which the instrument comprehensively represents the defined content domain. Quantitatively, item‐level content validity indices were calculated to assess each items's relevance and representativeness in measuring the selected core concepts. For three experts, an item was considered acceptable if it achieved an I‐CVI of 1.00, indicating full agreement [40, 41]. At the scale level, an S‐CVI/Ave value of ≥ 0.90 was regarded as indicative of acceptable content validity [40, 42]. Items that fell below the threshold but were considered essential for comprehensive construct coverage based on their conceptual representativeness were retained following consensus discussion [42, 43].

### 2.5 Pilot Survey Participant Recruitment

The pilot PCI was administered via the Qualtrics platform at the Faculty of Pharmacy and Pharmaceutical Sciences, Monash University. At the beginning of scheduled class and workshop sessions, the research team delivered a brief 10‐min presentation outlining the purpose of the study and the procedure for completing the pilot, after which students were invited to participate. Recruitment occurred through in‐class announcements, and students received an explanatory statement and consent form detailing the project's objectives, eligibility criteria, potential risks, and confidentiality provisions. Each cohort took the test across multiple separate sessions that students completed in a single sitting. The full set of items was completed as one test and was administered at the end of the year. Only students who had learned core pharmacology concepts as part of their coursework were eligible. Participation was entirely voluntary, carried no academic consequences, and was unrelated to assessment. No incentives were provided, and students were reminded they could withdraw at any time without penalty.

Pilot cohorts were selected conveniently through voluntary participation. Multiple cohorts at different stages of training were included to evaluate how the PCI items functioned across a range of learner groups. Their participation enabled analysis of item behavior, structure, and performance, contributing important learner perspectives to the initial validation and refinement of the PCI. The pilot was purely for research; the results were not used for feedback or assessment. Participants had completed pharmacology coursework 1–3 years prior, allowing assessment of both short‐ and long‐term conceptual recall and application.

### 2.6 Psychometric Analysis

Following the pilot survey, a psychometric evaluation of the PCI was undertaken to examine validity and reliability [44, 45]. Classical test theory was used as the primary framework [46, 47] to evaluate item‐level metrics [48, 49]. These analyses followed the Standards for Educational and Psychological Testing [44, 50] and provided the psychometric evidence required to refine the PCI. Item performance and internal structure were evaluated using indices of item difficulty, discrimination, and internal consistency. Internal consistency reliability was assessed using Cronbach's alpha. Item difficulty indicates how easy or challenging an item is, calculated as the proportion of students who answer correctly (*p*‐value, ranging from 0.0 to 1.0), and is classified as easy (*p* > 0.70), moderate (0.30 ≤ *p* ≤ 0.70), or difficult (*p* < 0.30). Items were flagged for revision if they were too easy (*p* > 0.80) or too hard (*p* < 0.20), as these items exhibiting ceiling or floor effects provide limited insights into students' abilities [51]. Item discrimination measures how effectively an item differentiates between high‐ and low‐performing students. This is typically assessed using the point‐biserial correlation or a simple discrimination index (values from −1.0 to 1.0). Discrimination is rated as excellent (≥ 0.40), good (0.30–0.39), fair (0.20–0.29), or poor (< 0.20) [52, 53]. Ideally, all items should distinguish between students who understand most concepts and those who do not. Items with moderate difficulty and strong discrimination were retained, even if slightly outside preferred ranges, due to their instructional relevance and alignment with common misconceptions.

## 3 Results

### 3.1 Demographics of Respondents

Seventy students completed the pilot PCI survey, with 70% (*n* = 49) enrolled in undergraduate programs and 30% (*n* = 21) in postgraduate studies (Table 1). Throughout their three to four years of study, students covered a variety of pharmacology topics, including pharmacokinetics, toxicology, advanced pharmacology, and pharmaceutical science, ensuring they had prior exposure to relevant concepts. On average, participants took approximately 40 min to complete the pilot.

**TABLE 1:** Demographic characteristics of respondents on pilot survey.

Theme	Categories	*N*	%
Program	**Undergraduate**	49	70
	BPharmSci (Hons)	21	30
	BPharm (Hons)	17	24
	BPharmSci Adv (Hons)	5	7
	Other (not specified)	6	9
	**Postgraduate**	21	30
	MPharmSci	16	23
	PhD (Pharmacology)	1	1
	MSc (Pharmacology)	2	3
	Other (not specified)	2	3
Year study	First year	15	21
	Second year	46	67
	Third year	9	13
Language^a^	English	31	44
	Chinese	23	33
	Arabic	5	7
	Indonesian	2	3
	Other (Amharic, Bahasa Indonesia, Dutch, German, Hindi, Hmong, Korean, Thai, Vietnamese)	9	13

Abbreviations: BPharm (Hons), Bachelor of Pharmacy (Honors); BPharmSci (Hons), Bachelor of Pharmaceutical Science (Honors); BPharmSci Adv (Hons), Bachelor of Pharmaceutical Science (Advanced Honors); MPharmSci, Master of Pharmaceutical Science.

^a^ Language demographics represent respondents' self‐identified first language. All surveys were administered in English.

### 3.2 PCI Item Construction

**FIGURE 2:**
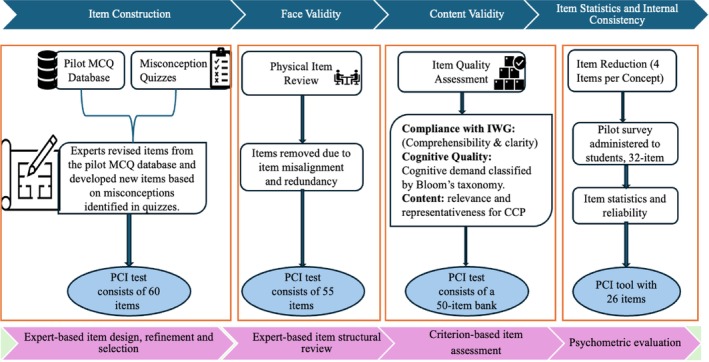
Conceptual overview of pharmacology concept inventory (PCI) item construction and validation methodology. CCP, Core Concepts of Pharmacology; IWG, Item Writing Guidelines; MCQ, Multiple Choice Question; PCI, Pharmacology Concept Inventory.

To ensure comprehensive representation of the identified core pharmacology concepts, eight international expert groups were organized, with each assigned to one core concept. Working collaboratively, the groups developed items by adapting questions from an existing pilot MCQ database and by constructing new items informed by misconceptions previously identified [26, 27, 30]. A minimum of six MCQs per core concept were constructed, yielding an initial pool of 60 items that targeted fundamental PK and PD concepts or sub‐concepts. After quality evaluations and multiple rounds of validation, this pool was refined to a final set of 26 PCI items (Figure 2).

### 3.3 Item Quality Evaluation

Following item construction, members of the expert educator team participated in the evaluation process after anonymous coding and categorization of all items to minimize potential bias. Three experts per item conducted an item‐level quality review to evaluate the content validity of the PCI. Each expert assessed only items that they had not authored, thereby maintaining neutrality. Face validity assessment was performed to ensure alignment with the intended core concept and to identify item content duplication. Five items that were redundant or poorly aligned with the core concepts were removed, resulting in a pool of 55 items (Table 2). The remaining items were then evaluated through a structured content quality assessment using QAF.

**TABLE 2:** Item compliance with item‐writing guidelines (IWG) and item‐level content validity index (I‐CVI).

Pharmacokinetics domain items			Pharmacodynamics domain items		
Items	Compliance level	Core concept assessment	Items	Compliance level	Core concept assessment
	Median (IQR)	I‐CVI		Median (IQR)	I‐CVI
DCl1	0.91 (0.07)	0.33^a^	DE1	0.91 (0.07)	1.00
DCl2^d^	0.91 (0.09)	0.67^b^	DE2	0.96 (0.00)	1.00
DCl3	0.91 (0.02)	0.33^a^	DE3	0.96 (0.00)	1.00
DCl4^d^	0.96 (0.02)	0.33^a^	DE4	0.96 (0.02)	1.00
DCl5^d^	0.91 (0.04)	1.00	DE5	0.91 (0.02)	1.00
DCl6^d^	0.91 (0.02)	1.00	DE6	0.91 (0.04)	1.00
DBio1	1.00 (0.00)	0.67^b^	DE7	0.96 (0.02)	1.00
DBio2	1.00 (0.00)	1.00	DT1	0.96 (0.04)	1.00
DBio3	1.00 (0.00)	1.00	DT2	1.00 (0.02)	1.00
DBio4	0.91 (0.07)	1.00	DT3	0.91 (0.02)	1.00
DBio5	1.00 (0.00)	1.00	DT4	0.91 (0.02)	1.00
DBio6	0.96 (0.07)	1.00	DT5	0.91 (0.04)	0.67^b^
DBio7	1.00 (0.00)	1.00	DT6	1.00 (0.02)	1.00
DBio8	1.00 (0.02)	1.00	DT7	0.96 (0.02)	1.00
DBio9	0.83 (0.09)	1.00	DTI1	0.96 (0.04)	1.00
Css1	0.91 (0.09)	1.00	DTI2	1.00 (0.00)	1.00
Css2	0.83 (0.13)	1.00	DTI3	0.96 (0.04)	1.00
Css3	0.78 (0.09)	1.00	DTI4	0.91 (0.09)	0.00^c^
Css4	0.91 (0.07)	0.67^b^	DTI5	0.96 (0.13)	1.00
Css5	0.96 (0.02)	1.00	DTI6	1.00 (0.02)	1.00
Css6	1.00 (0.00)	1.00	DTI7	0.91 (0.07)	0.33^a^
Css7	0.96 (0.02)	1.00	SAR1	0.83 (0.04)	0.67^b^
Css8	0.96 (0.00)	1.00	SAR2	0.87 (0.13)	0.67^b^
Vd1	0.96 (0.20)	0.67^b^	SAR3	0.87 (0.13)	0.67^b^
Vd2	0.96 (0.09)	1.00	SAR5	0.78 (0.11)	1.00
Vd3	1.00 (0.04)	1.00	SAR6	0.70 (0.11)	0.33^a^
Vd4	0.91 (0.20)	1.00			
Vd5	0.96 (0.11)	1.00			
Vd6	0.91 (0.22)	1.00			

*Note:* Scale‐level content validity indices (S‐CVI/Ave) for 55 items = 0.87; for 50 items = 0.93.

Abbreviations: Css, steady‐state concentration; DBio, drug bioavailability; DCI, drug clearance; DE, drug efficacy; DT, drug tolerance; DTI, drug target interaction; I‐CVI, item‐level content validity index; IQR, interquartile range; SAR, structure–activity relationship; Vd, volume of distribution.

^a^ Require significant improvement.

^b^ Require moderate improvement.

^c^ Candidate for modification or deletion. Adherence Level: 0 = not adhering to any of the IWGs; 1 = adhering to all IWGs.

^d^ For the next version of the test (shown on Table 3, items 32 and 26), the following recoding was performed: DCl2 → DCl1; DCl4 → DCl2; DCl5 → DCl3; DCl6 → DCl4.

The PCI tool was assessed for three quality dimensions: clarity and structure of item writing; cognitive levels assessed; and relevance to and representation of the target core concept. The quality of item writing, as determined by adherence to the item‐writing guidelines, resulted in 12 items rated high, 41 good, and 2 fair. Overall, adherence showed a high median (interquartile range (IQR)) value ranging from 0.70 (0.11) to 1.00 (0.02) (Table 2). Regarding cognitive domain classifications, 19 items assessed application, 20 focused on comprehension, 14 addressed knowledge, and 2 targeted analysis. This distribution reflects an emphasis on understanding and application, with a particular focus on higher‐order thinking skills (Figure S3). Finally, the item pool was assessed for relevance and effectiveness, and 25 items covered the main pharmacology concepts, another 25 tested on sub‐concepts, and 5 targeted minor or inadequately addressed core concepts.

### 3.4 Content Validity

Relevance and representativeness of PCI items in addressing the intended core concepts were further examined using the item and scale level content validity indices (I‐CVI and S‐CVI), which are used to quantify experts' agreement on whether items accurately reflect the construct. A high level of agreement (I‐CVI = 1.00) was observed for three‐quarters of the items (*n* = 41), moderate consensus (I‐CVI = 0.67) for eight questions, and low agreement (I‐CVI = 0.33) for the remaining (*n* = 6) items. Based on these findings, five items with I‐CVI scores below 0.67 were excluded, namely drug clearance (3 items), drug–target interaction (2 items), and structure–activity relationships (1 item). Items with lower specified indices were removed from the item set; however, one drug clearance item with a lower I‐CVI of 0.33 was retained following expert consensus, as it was considered conceptually relevant and representative. Consequently, the final item bank comprised 50 MCQs deemed appropriate for assessing the main or sub‐concepts of pharmacology.

The overall scale‐level content validity index using the average method (S‐CVI/Ave) increased from 0.87 to 0.93 after removing low‐validity items, indicating improved overall content validity (Table 2). To optimize test‐takers' participation and maximize the test completion rate, a 32‐item version of the instrument (22 focused on the main core concepts and 10 targeted at sub‐concepts) was administered for the first pilot survey. Items were selected based on their relevance to and representativeness of the core concepts, with four items for each core concept retained for piloting, while remaining items were kept in an item bank for subsequent testing.

### 3.5 Conceptual Understanding and Misconception Patterns on Core Concepts

We analyzed the distribution of responses for each PCI item, categorizing them as correct, distractor, or uncertain. Across PK questions, students answered a mean (standard deviation (SD)) of 46% (SD = 13) correctly. For the remaining responses, approximately 39% (SD = 12) were distractors, and 16% (SD = 8) were uncertain responses. In the PD domain, performance was slightly higher, with students correctly answering a mean of 50% (SD = 10) of responses. Distractor responses decreased to 31% (SD = 11), while uncertainty increased to 19% (SD = 7). When combining PK and PD items, mean (SD) response rates were 48% correct (SD = 12), 35% distractors (SD = 12), and 17% uncertain (SD = 8). Among core concepts, the mean proportion of correct responses ranged from 41% for volume of distribution to 52% for drug tolerance. Drug bioavailability, drug efficacy, and drug‐target interaction concepts each showed an average of correct response rates of 50%, whereas structure–activity relationship, steady‐state concentration, and drug clearance concepts ranged from 45% to 49%.

**FIGURE 3:**
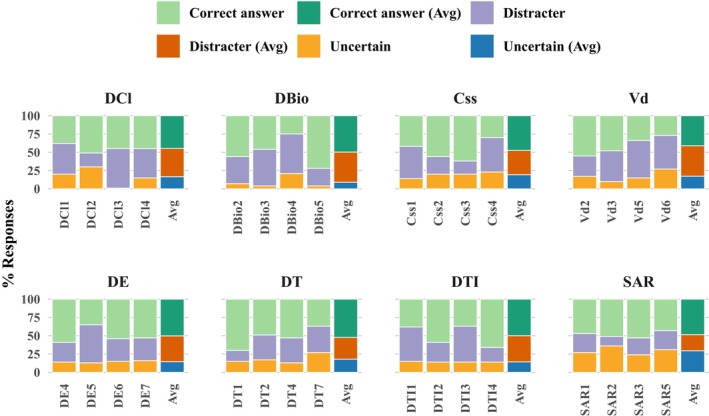
Distribution of student response accuracy by item across pharmacological core concepts. Css, Steady state concentration; DBio, Drug bioavailability; DCl, Drug clearance; DE, Drug efficacy; DT, Drug tolerance; DTI, Drug–target interactions; SAR, Structure–activity relationships; Vd, Volume of distribution.

Variation was also observed at the item level within concepts, with correct response rates ranging from 25% to 72% for drug bioavailability, 30% to 62% for steady‐state concentration, 37% to 70% for drug tolerance, and 37% to 66% for drug‐target interaction. Additionally, uncertainty levels varied widely across concepts, ranging from 30% for the structure–activity relationship items to 9% for drug bioavailability, which had the lowest proportion of uncertain responses. Across most core concepts, students achieved more than 50% correct responses on two of the four items within each concept. Performance was strongest for drug efficacy, with three of the four items exceeding 50% correct responses. In contrast, drug tolerance, drug bioavailability, steady‐state concentration, drug–target interactions, and structure–activity relationships each showed two of the four items with more than 50% correct responses. Drug clearance and volume of distribution were more challenging, with only one of the four items in each concept reaching the 50% correct responses (see Figure 3).

### 3.6 Item Statistics and Reliability

Classical test theory was employed, and item statistics along with reliability (Cronbach's alpha) metrics were obtained across two item sets: an initial item set of 32 items and a final set of 26 items. Based on the analysis, four items: one drug bioavailability item, two drug–target interaction items, and one volume of distribution item were removed because they showed low discrimination indices, and their removal improved the overall Cronbach's alpha values. Additionally, two drug–target interaction items demonstrated low reliability and indicated the need for more items to support the drug‐target interaction concept. Due to this limitation, the drug‐target interaction concept was excluded from further analysis (Table 3).

**TABLE 3:** Item statistics and reliability metrics across two item sets per core concept.

Items	Survey test version (32 items)			Refined test version (26 items)		
	Item difficulty_1_	Item discrimination_1_	Cronbach alpha (α_1_)	Item difficulty_2_	Item discrimination_2_	Cronbach alpha (α_2_)
Css1	0.43	0.37	0.65	0.43	0.37	0.65
Css2	0.56	0.39		0.56	0.39	
Css3	0.61	0.46		0.61	0.46	
Css4	0.3	0.51		0.3	0.51	
DBio2	0.57	0.35	0.56	0.57	0.37	0.64
DBio3	0.47	0.38		0.47	0.42	
DBio4	0.24	0.12^a^		^c^	^c^	^c^
DBio5	0.71	0.54		0.71	0.56	0.64
DCl1	0.39	0.44	0.65	0.39	0.44	0.65
DCl2	0.51	0.36		0.51	0.36	
DCl3	0.46	0.53		0.46	0.53	
DCl4	0.46	0.4		0.46	0.4	
DE4	0.6	0.55	0.68	0.6	0.55	0.68
DE5	0.34	0.43		0.34	0.43	
DE6	0.53	0.45		0.53	0.45	
DE7	0.53	0.42		0.53	0.42	
DT1	0.71	0.54	0.75	0.71	0.54	0.75
DT2	0.5	0.58		0.5	0.58	
DT4	0.53	0.57		0.53	0.57	
DT7	0.37	0.47		0.37	0.47	
DTI1	0.37	0.09^a^	0.37	^c^	^c^	^c^
DTI2	0.59	0.28^b^				
DTI3	0.37	0.03^a^				
DTI4	0.66	0.46^b^				
SAR1	0.47	0.74	0.85	0.47	0.74	0.85
SAR2	0.51	0.63		0.51	0.63	
SAR3	0.53	0.63		0.53	0.63	
SAR5	0.43	0.75		0.43	0.75	
Vd2	0.56	0.51	0.61	0.56	0.49	0.68
Vd3	0.47	0.49		0.47	0.57	
Vd5	0.34	0.19^a^		^c^	^c^	^c^
Vd6	0.26	0.41		0.26	0.44	0.68

*Note:* Numbers following each core concept label indicate the item number assigned to that concept (e.g., Css1–Css4).

Abbreviations: Css, Steady state concentration; DBio, Drug bioavailability; DCl, Drug clearance; DE, Drug efficacy; DT, Drug tolerance; DTI, Drug–target interactions; SAR, Structure–activity relationships; Vd, Volume of distribution.

^a^ Items with a lower discrimination index.

^b^ Items with poor reliability (higher alpha values, when the item is deleted).

^c^ Items removed from the refined test version (26 items).

**FIGURE 4:**
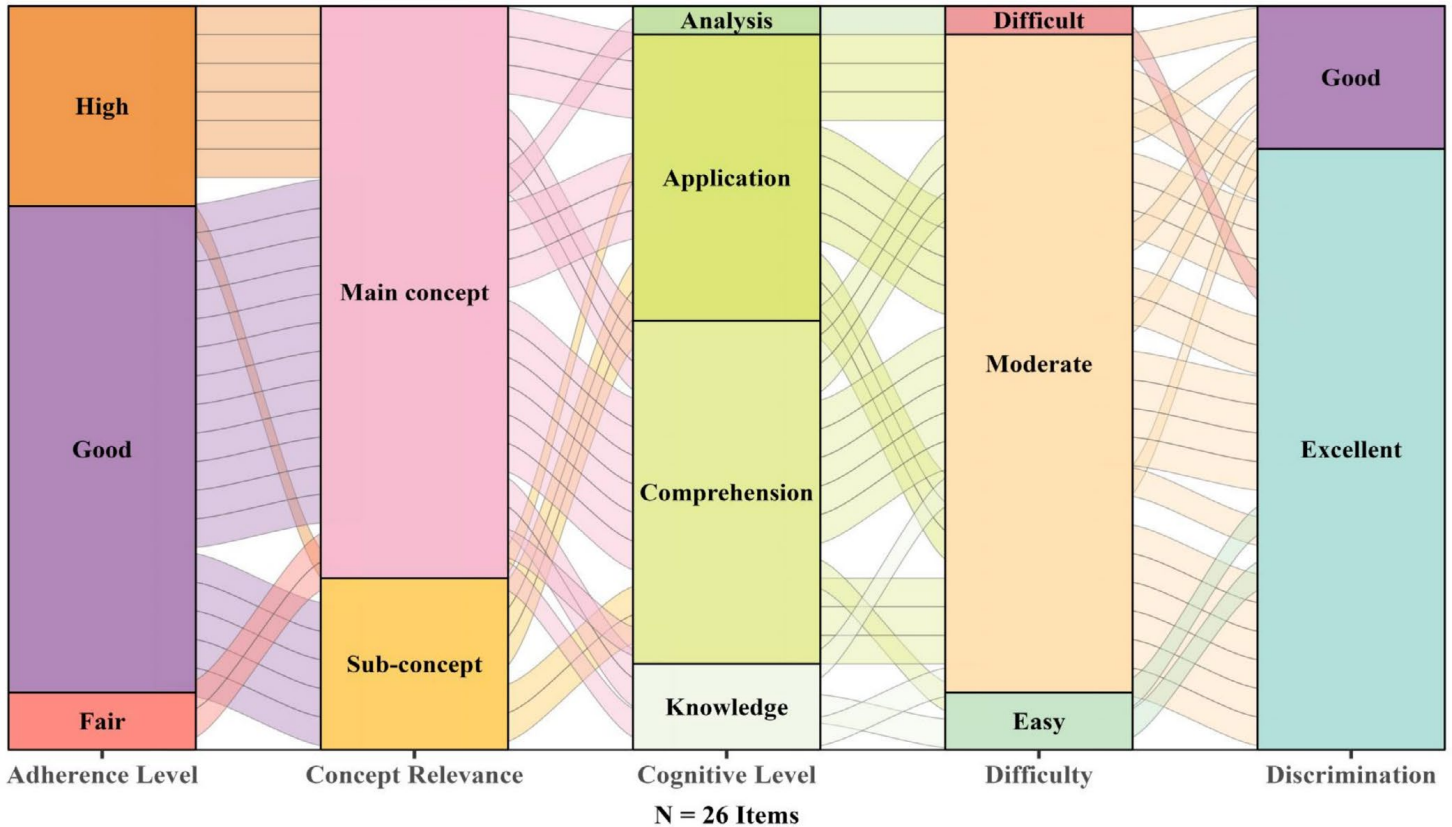
Mapping of PCI tool item quality and statistical performance metrics. Alluvial plot illustrating how 26 items transition across five dimensions: adherence level to item writing guidelines (High, Good, and Fair), concept relevance (Main concept, Sub‐concept), and cognitive demand based on Bloom's taxonomy (Analysis, Application, Comprehension, and Knowledge), challenge level (Difficult, Moderate, and Easy) and degree of discrimination (Good, Excellent).

Following removal of these items, the final 26 items underwent evaluation across five dimentions of item quality: adherence level to guidelines, concept relevance, cognitive level, item statistics and reliability. Most items (*n* = 24) demonstrated high or good adherence to the guidelines, while the remaining (*n* = 2) showed fair compliance. About three‐quarters of the items (*n* = 20) targeted the main pharmacology core concept, with the remaining (*n* = 6) assessing sub‐concepts. Regarding the cognitive level, most items were concentrated in mid‐ to high‐order categories of Bloom's taxonomy, with 12 items assessing comprehension, 10 items addressing the application level, one item targeting analysis and three items addressing knowledge (Figure 4).

In addition to these qualitative characteristics, the items were also examined for statistical properties. Most items had moderate difficulty indices (*n* = 23), and questions with better recommended difficulty levels were more likely to demonstrate excellent and good discrimination levels, indicating their effectiveness in identifying different levels of students' performance. Furthermore, the overall internal consistency of the PCI was high, with a Cronbach's alpha of 0.91. The Cronbach's alpha values at the core concept level ranged from 0.64 to 0.85. Most item sets had moderate to acceptable internal consistency (α = 0.64–0.75), while two concepts had high reliability (α = 0.74–0.85) (Table 3).

## 4 Discussion

This study developed and validated a PCI tool to assess students' conceptual understanding and identify the prevalence of documented misconceptions in concept‐based pharmacology education. It also aimed to generate insights that can directly inform curriculum development by highlighting areas requiring targeted instructional support, guiding the sequencing of content, and enabling evidence‐based refinement of teaching strategies. The work extends earlier phases of the IUPHAR‐Ed Core Concepts of Pharmacology Project [28, 29], which established a consensus framework of core pharmacology concepts. To ensure robustness, we triangulated theoretical frameworks [34, 47], expert input, and student perspectives [3].

This study's findings showed that item quality in the PCI is a multidimensional construct, characterised through a structured evaluation framework [30]. Items were deemed high‐quality when they demonstrated conceptual alignment with Core Concepts of Pharmacology [28, 29], addressed appropriate cognitive levels based on Bloom's taxonomy [37], adhered to item‐writing guidelines that ensured clarity of stems, plausibility of distractors, and minimised construct‐irrelevant cues [2, 54], and acceptable psychometric performance, including item difficulty and discrimination, and reliability, in accordance with classical test theory [46, 47, 55, 56]. The reduction from 60 initial items to 26 retained reflects rigorous empirical curation rather than widespread item failure, with exclusions mainly due to item repetition, construct overlap, and feasibility considerations or the need to limit student burden. The remaining items will be refined, used for parallel‐test development, or undergo ongoing validation, following best assessment practices [57]. Consistent with these findings, the study suggests that maximizing item retention in future item banks depends on early conceptual specification, expert calibration, and the incorporation of student‐derived misconceptions during item development, rather than post hoc statistical elimination. Together, these results provide practical guidance for pharmacology educators by demonstrating that sustainable, concept‐focused assessment prioritises diagnostic value over sheer item quantity.

Involvement of international pharmacology educators from ten countries across four continents in constructing and evaluating of the item bank ensured that the PCI tool was both conceptually robust and pedagogically relevant. The quality and applicability of concept inventories were grounded in authentic disciplinary reasoning rather than superficial recall tasks [15, 58]. Guided by structured item writing principles, experts improved the quality and conceptual accuracy of items, which may help test‐takers better understand key concepts during assessment of learning gains and identify documented misconceptions. Compliance with guidelines can improve concept inventory tools to align with specific conceptual domains, maintaining item independence, concise stems, plausible distractors and clear language, thereby minimizing ambiguity, bias, and unintended clues, while enhancing cognitive rigor and fairness [2]. Besides this structural strength, assessing cognitive depth was crucial to designing the inventory. Creating items in line with Bloom's taxonomy levels may increase the tool's ability to assess intended learning outcomes and evaluate clinical decision‐making competencies [37]. The PCI mainly addresses comprehension and application domains, underscoring its ability to evaluate higher‐order thinking, thereby enhancing both its diagnostic and educational utility. These evaluations strengthen the PCI's ability to assess concept‐based pharmacology learning [28, 29] in real‐world contexts, reinforcing its dual role as a diagnostic and educational tool that fosters concept‐based learning and conceptual understanding.

An essential aspect of inventory development is establishing content validity, which ensures that items are relevant and accurately represent the intended conceptual domain. Concept inventories are meaningful only if they effectively measure the concepts they aim to assess while excluding irrelevant or minor elements [50]. In the PCI, expert‐driven design and iterative review improved both quality and representation by refining and retaining items aligned with the concept while removing unrelated ones. The current study provided strong evidence of content validity, showing high expert agreement on most items and consistently high scores at both item and scale levels. These results position the PCI as a tool capable of assessing pharmacologic understanding beyond simple recall, offering valuable insights for measuring learning outcomes and guiding instruction. Importantly, this expert validation aligns with best practices in concept inventory development [5, 13], in which thorough review processes support the long‐term credibility and impact of these instruments.

Following the establishment of content validity, the psychometric testing of the PCI provides strong evidence that the instrument performs reliably and aligns with the conceptual intentions of its design. The high internal consistency (α = 0.91) indicates that the items collectively capture a coherent representation of the targeted pharmacological concepts, suggesting that students' responses reflect systematic variations in conceptual understanding rather than random guessing. This level of reliability is consistent with benchmarks for established concept inventories [5, 44], in which higher scores are typically interpreted as evidence of stable construct measurement [44, 50]. Furthermore, the acceptable‐to‐strong reliability across most conceptual categories supports the PCI's capacity to provide consistent inferences across subdomains, an essential feature for domain‐specific diagnostic tools.

The item discrimination indices further affirm the tool's diagnostic precision. Items that effectively differentiate higher‐ from lower‐performing students are considered vital to conceptual assessments because they indicate that performance reflects understanding rather than test‐taking ability [52, 53]. The tool's discrimination pattern suggests that its items are well‐calibrated to reveal differences in conceptual mastery of core concepts. Similarly, the predominance of items with moderate difficulty values aligns with psychometric recommendations that concept inventories should avoid ceiling or floor effects to ensure sensitivity across ability levels [52, 53]. Importantly, integrating these quantitative indicators with prior expert validation underscores that the instrument's statistical robustness complements its conceptual representativeness, together strengthening the validity argument for its use as a research and instructional diagnostic tool.

The refinement of the PCI through systematic item removal highlights the crucial role of psychometric filtering in developing a valid and reliable assessment tool. Excluding items with weak discrimination or conceptual misalignment improved internal consistency and content validity. Item reduction is not a limitation but rather a necessary stage in developing the instruement, ensuring that the retained items are educationally meaningful and statistically acceptable. Comparable iterative refinements have been central to the development of established concept inventories, for which the removal of low‐performing items was essential for establishing credibility and applicability [44, 52, 53]. For the PCI, this refinement underscores its robustness as a balanced instrument that integrates comprehensive coverage of core pharmacology with psychometric rigor, ultimately strengthening its utility for assessing student understanding and measuring the prevalence of misconceptions.

Alongside psychometrically validated item refinement, item‐level response distributions within the PCI can provide insights into diagnosing cognitive frameworks. Higher selection frequencies for particular distractors or response uncertainties may indicate concepts linked to systematic misconceptions or incomplete conceptual understanding. This response pattern provides empirical feedback for instructors by highlighting learning challenges that could be addressed through targeted, concept‐specific instructions or item refinement. Responses across concept domains showed that PD items elicited slightly higher rates of correct answers and fewer incorrect selections than PK items, but were associated with greater uncertainty. This pattern suggests that students may hold varied understandings and perform differently across domains. Moreover, accuracy varied across concepts, with the strongest and most consistent performance observed for drug tolerance and drug efficacy, lower achievement for volume of distribution and drug clearance, and uncertainty concentrated in structure–activity, reflecting uneven mastery of key concepts. At this stage, incorrect responses should be interpreted as either distractors or uncertainties rather than definitive misconceptions. Consequently, follow‐up interviews and an analysis of the open‐ended explanations are essentail to distinguish authentic conceptual misunderstandings from random errors, thereby guiding improvements in assessment designs and teaching approaches.

While concept inventories are evidence‐based tools employed for various purposes, their interpretation warrants caution due to inherent limitations. Inventories have recognized constraints in fully capturing the complexity of students' reasoning. Responses may be influenced by item wording, contextual cues, or surface features rather than by the intended conceptual reasoning, while ambiguous phrasing and discipline‐specific terminology may further confound interpretation, particularly for students less familiar with the concept domain [58]. Additionally, the multiple‐choice format permits random or strategic guessing; correct answers may reflect partial knowledge, while incorrect ones may stem from a limited background rather than misconceptions [59]. Inclusion of justification tiers could help to identify guessing from consistently held misconceptions and guide targeted remediation planning [18], though some item interpretation ambiguities may still exist.

## 5 Educational Significance

The PCI can support concept‐based pharmacology education by guiding the assessment of conceptual understanding, evaluating learning outcomes, and informing instruction. Grounded in established learning theories and informed by expert viewpoints and student perspectives, it provides insights into learners' cognitive processes and promotes the adoption of learner‐centered instructional approaches, which are essential for pharmacology training. By integrating expert review, empirical psychometric evidence, and student‐driven distractors, the PCI captures how learners apply core pharmacology principles in reasoning rather than simple recall. This design enhances its diagnostic usefullness for educators and researchers, encouraging concept‐focused curricula and evidence‐based assessment practices.

The PCI is designed for flexible implementation across instructional contexts, consistent with established use of concept inventories, particularly in STEM education [3, 5]. Prior studies demonstrate that concept inventories are commonly administered both formatively, as diagnostic pre‐tests, mid‐course assessments, or topic‐level probes to identify misconceptions and guide teaching, and summatively to evaluate conceptual mastery, learning gains, curriculum effectiveness, and cohort‐level outcomes following instruction [13, 14, 15]. Accordingly, the PCI may be implemented before, during, or after a course depending on pedagogical intent. The educational impact of PCI use is therefore contingent on its instructional integration, with formative and summative applications serving complementary roles in feedback, evaluation, and ongoing instrument validation.

The development of the PCI can provide valuable insights for educators aiming to create high‐quality, content‐specific assessment items. Effective questions were developed through explicit specification of the target concepts, alignment with established item‐writing guidelines, and mapping to appropriate cognitive levels to elicit conceptual reasoning rather than factual recall [2, 37]. Incorporating learner‐derived misconceptions alongside expert‐led refinement was particularly effective for generating plausible, diagnostically meaningful distractors, a key indicator of high‐quality items [52, 60]. However, this process required structured consensus to maintain conceptual coherence. Iterative refinement using both psychometric evidence and response‐process insights was critical in distinguishing what worked from what did not, as items developed without empirical evaluation often exhibited ambiguity, weak discrimination, or ineffective distractors.

## 6 Limitations and Future Work

This pilot study, conducted with a small sample from a single institution, may limit the representativeness and broad applications of the findings. Differences in course design and teaching approaches across institutions could influence how well the current instrument performs in various curricular contexts. As the Core Concepts of Pharmacology Project seeks to align pharmacology curricula, addressing these contextual variations will be a key focus moving forward. Currently, data on students' conceptual understanding, misconceptions, and psychometric testing are preliminary, and the small sample size has limited comparisons between different cohorts. Additionally, the PCI was administered as a voluntary, non‐summative research tool, which might have lowered student motivation and preparation compared to summative assessments. Variations in the timing of assessments relative to prior pharmacology instruction could also affect recall: tests administered closer to the content delivery might benefit from short‐term memory, whereas those given after longer intervals might not.

The current PCI covers seven of the 24 core concepts, with additional items planned for subsequent phases. The next phase of the PCI project will collect open‐ended responses and conduct interview analyses to gather response process validity, which will further guide in identifying misconceptions and assessing conceptual reasoning to support PCI validation and refinement. Future administration will align PCI application with educational goals, adapting testing conditions and timing relative to instruction for formative or summative purposes to enable accurate interpretation of results. This approach may lead to a more precise interpretation of test results and evaluate motivation‐dependent and long‐term retention effects.

Expert‐led revisions and large‐scale international surveys will follow, supported by ongoing psychometric evaluation at each administration. This iterative testing will allow monitoring of item performance across different cohorts with varying levels of competence and in both summative and formative contexts, enabling for refinement of items as educational emphasis and program integration evolve. Continuous psychometric evaluation will support PCI development stability and enhance the evidence‐based assessment effectiveness of concept‐based pharmacology education.

## 7 Conclusion

The development and initial validation of the PCI tool integrated educational theory, educator insights, and learner perspectives to ensure conceptual and methodological rigor. This triangulated approach produced a promising assessment tool capable of evaluating conceptual understanding, measuring the prevalence of misconceptions, and measuring learning outcomes. Expert review confirmed item quality and content validity, while pilot testing provided evidence of item performance and internal structure across the core concepts of pharmacology. By revealing students' strengths and gaps in complex topics, the PCI supports targeted, concept‐focused learning strategies. Further, content and qualitative analysis of interview responses offer insight into reasoning processes and misconceptions in critical concepts. These findings can inform curriculum refinement and guide evidence‐based teaching approaches. Overall, the PCI represents a valuable resource for assessing learning and enhancing instructional design in pharmacology education.

## Author Contributions

**Adeladlew K. Netere:** conceptualization, methodology, software, data curation, investigation, validation, formal analysis, visualization, project administration, resources, writing – original draft, writing – review and editing. **Tony Hughes:** conceptualization, investigation, methodology, project administration, visualization, writing – original draft, writing – review and editing. **Anna‐Marie Babey:** conceptualization, data curation, formal analysis, investigation, methodology, visualization, validation, writing – original draft, writing – review and editing. **Clare Guilding:** conceptualization, investigation, methodology, visualization, validation, writing – original draft, writing – review and editing. **Carolina Restini:** conceptualization, data curation, investigation, validation, methodology, visualization, writing – original draft, writing – review and editing. **Martin Hawes:** conceptualization, investigation, methodology, visualization, writing – original draft, writing – review and editing. **John P. Kelly:** conceptualisation, investigation, methodology, visualization, writing – original draft, writing – review and editing. **Elvan Djouma:** conceptualisation, investigation, methodology, visualization, writing – original draft, writing – review and editing. **Jennifer Koenig:** conceptualisation, formal analysis, investigation, methodology, visualization, writing – original draft, writing – review and editing. **Jacqueline E. McLaughlin:** conceptualisation, formal analysis, investigation, methodology, visualization, writing – original draft, writing – review and editing. **Olusola Olafuyi:** conceptualisation, formal analysis, investigation, methodology, visualization, writing – original draft, writing – review and editing. **Lynette B. Fernandes:** conceptualisation, formal analysis, investigation, methodology, visualization, writing – original draft, writing – review and editing. **Janet Mifsud:** conceptualisation, investigation, methodology, visualization, writing – original draft, writing – review and editing. **Graeme J. Sills:** conceptualisation, investigation, validation, visualization, writing – original draft, writing – review and editing. **Anneke H. van Houwelingen:** formal analysis, investigation, methodology, validation, visualization, writing – original draft, writing – review and editing. **Steven J. Tucker:** investigation, methodology, visualization, validation, writing – original draft, writing – review and editing. **Willmann Liang:** formal analysis, investigation, validation, methodology, visualization, writing – original draft, writing – review and editing. **Patrik Aronsson:** conceptualisation, investigation, methodology, visualization, writing – original draft, writing – review and editing. **Farhan Ahmad Khan:** investigation, methodology, visualization, validation, writing – original draft, writing – review and editing. **Tina Hinton:** conceptualisation, formal analysis, investigation, methodology, visualization, writing – original draft, writing – review and editing. **Mark Hernandez:** conceptualisation, formal analysis, investigation, methodology, visualization, writing – original draft, writing – review and editing. **Lindsay Cormier:** conceptualisation, investigation, methodology, visualization, writing – original draft, writing – review and editing. **Roisin Kelly‐Laubscher:** conceptualisation, formal analysis, investigation, methodology, project administration, visualization, writing – original draft, writing – review and editing. **Fabiana A. Caetano Crowley:** conceptualisation, data curation, formal analysis, investigation, methodology, visualization, writing – original draft, writing – review and editing. **Marina Junqueira Santiago:** conceptualisation, investigation, visualization, validation, writing – original draft, writing – review and editing. **Margaret Cunningham:** conceptualisation, investigation, methodology, visualization, writing – original draft, writing – review and editing. **Jennelle Durnett Richardson:** conceptualisation, investigation, methodology, visualization, writing – original draft, writing – review and editing. **Kelly Karpa:** conceptualisation, investigation, visualization, writing – original draft, writing – review and editing. **Paul J. White:** methodology, software, investigation, validation, visualization, project administration, supervision, resources, writing – original draft, writing – review and editing.

## Conflicts of Interest

The authors declare no conflicts of interest.

## Supporting information


**Figure S1:** Core concepts of pharmacology included in the PCI instrument.

**Figure S2:** Sample item design structure and iterative refinement process on various phases (Css1, Steady State Concentration Concept item 1).

**Figure S3:** Mapping the 55‐item PCI test across the quality analysis framework. Alluvial plot illustrating how 55 assessment items transition across three dimensions: adherence level to item writing guidelines (High, Good, and Fair), relevance to the target construct (Main Concept, Sub‐Concept, Minor Concept, and Inadequate), and cognitive demand based on Bloom's Taxonomy (Analysis, Application, Comprehension, and Knowledge). Each block (strata) represents dimensions, and the flow is from adherence level ⟶ concept relevance ⟶ cognitive level. Flow width represents the *number of items* in that category combination.

**Table S1:** Pharmacology expert team composition for PCI tool item development and validation.

## Data Availability

All working data for this study will be available on reasonable request to the corresponding author.
