# Supplementary material for: Development and Validation of Pharmacology Concept Inventory for Concept‐Based Learning: Leveraging Theory, Expert Insights, and Student Perspectives
Source: Pharmacol Res Perspect. 2026 Mar 22;14(2):e70237. doi: 10.1002/prp2.70237 (PMC13140222; doi:10.1002/prp2.70237)
Supplement: Supplementary file 1 — Figure S1: Core concepts of pharmacology included in the PCI instrument. [file PRP2-14-e70237-s002.docx]

**Figure S1**: Core Concepts of Pharmacology included in the PCI instrument
