# Supplementary material for: Development and Validation of Pharmacology Concept Inventory for Concept‐Based Learning: Leveraging Theory, Expert Insights, and Student Perspectives
Source: Pharmacol Res Perspect. 2026 Mar 22;14(2):e70237. doi: 10.1002/prp2.70237 (PMC13140222; doi:10.1002/prp2.70237)
Supplement: Supplementary file 2 — Figure S2: Sample item design structure and iterative refinement process on various phases (Css1, Steady State Concentration Concept item 1). [file PRP2-14-e70237-s003.docx]

**Figure S2:** Sample item design structure and iterative refinement process on various phases (**Css1**: Steady State Concentration Concept item 1)

| **Phase** | **Item Structure** | **Rational Design Features** | **Purpose of Modification** |
| --- | --- | --- | --- |
| Phase 1: Initial item construction  (Single --Tier) | **Stem**  To treat an oral infection, the dose amount of an antibiotic was increased from 200 mg twice daily to 400 mg twice daily. Which one of the following is expected to be observed on the pharmacokinetic profile of the drug?  **Options**   1. The time to reach a steady state will increase 2. The time to reach a steady state will decrease 3. The time to reach a steady state will remain unchanged (correct) 4. The plasma concentration at a steady state will decrease | Distractors designed to reflect common identified misconceptions:   - Confusion between dose and half-life - Misaddressing of steady state determinants | Assess conceptual understudying of the relationship between dose, half-life and time to steady state |
| Explanation | **Correct answer- Option-C**  The time required to reach steady state concentration is dependent on the half-life. Increasing the dose amount for a repetitive dosing schedule increases the steady state C_min_ and C_max_ but does not increase the time taken to reach the steady. | Conceptual target: Half-life determines steady state timing | Ensure alignment with core pharmacokinetics principles |
| Phase 2: Refined version (Two-Tier) | **Added option**   1. I am unsure / I cannot answer this confidently.   Reasoning: “*Explain why you chose this option.*”: ___________________________________________ | “*Unsure*” to reduces random guessing; reasoning to evaluate justification for the selected answer | Improve diagnostic precision |
| Pilot survey  (Final version) | Same stem and options (A-E), and open-ended justification | Used in pilot survey | Enabled analysis of misconception pattern and reasoning alignment |

Sample item design structure and refinement process on various stages
