# Supplementary material for: Development and Validation of Pharmacology Concept Inventory for Concept‐Based Learning: Leveraging Theory, Expert Insights, and Student Perspectives
Source: Pharmacol Res Perspect. 2026 Mar 22;14(2):e70237. doi: 10.1002/prp2.70237 (PMC13140222; doi:10.1002/prp2.70237)
Supplement: Supplementary file 3 — Figure S3: Mapping the 55‐item PCI test across the quality analysis framework. Alluvial plot illustrating how 55 assessment items transition across three dimensions: adherence level to item writing guidelines (High, Good, and Fair), relevance to the target construct (Main Concept, Sub‐Concept, Minor Concept, and Inadequate), and cognitive demand based on Bloom's Taxonomy (Analysis, Application, Comprehension, and Knowledge). Each block (strata) represents dimensions, and the flow is from adherence level ⟶ concept relevance ⟶ cognitive level. Flow width represents the number of items in that category combination. [file PRP2-14-e70237-s001.docx]

**
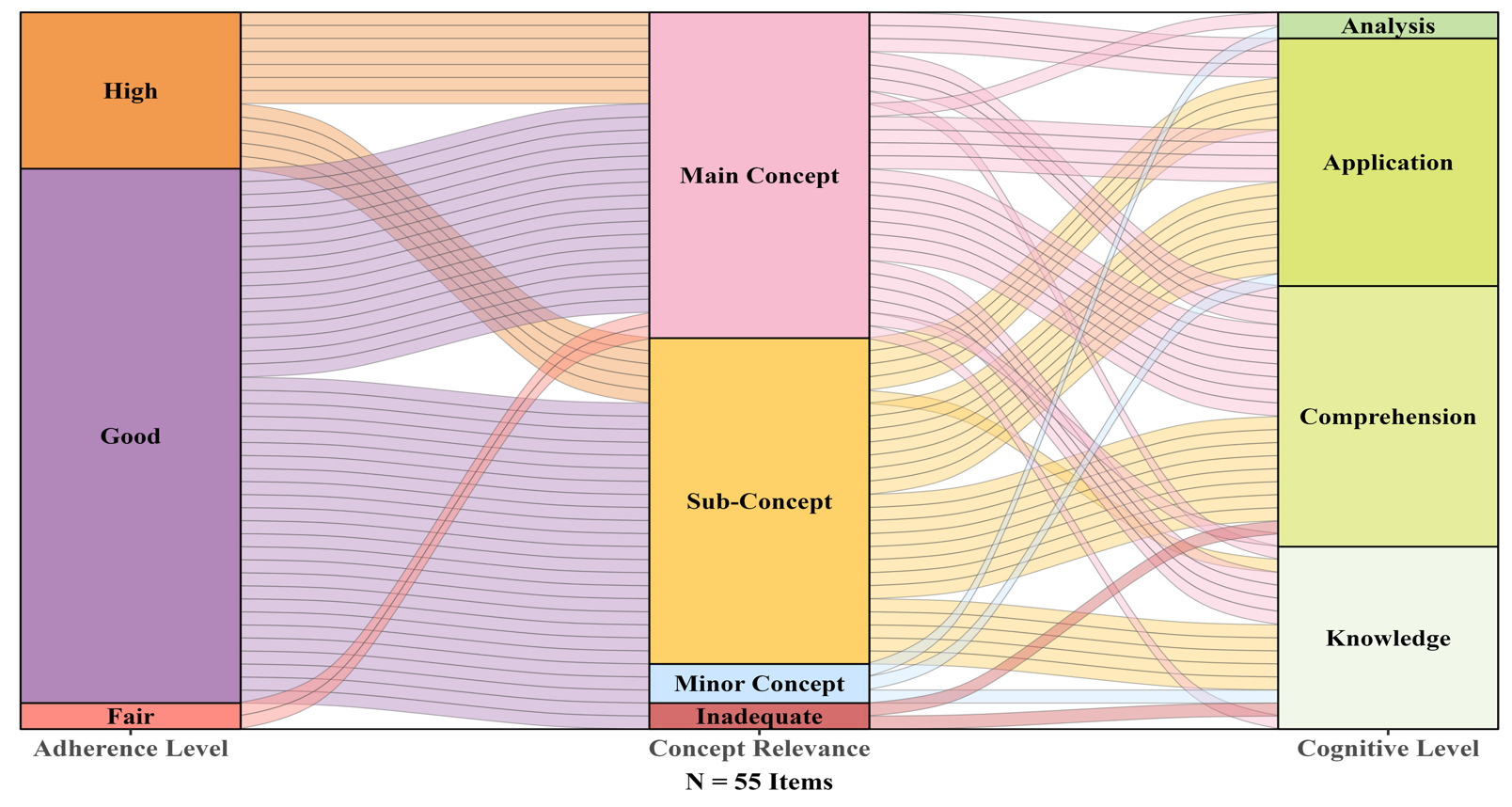
**

**Figure S3**: Mapping the 55-item PCI test across the quality analysis framework

**NB:** Alluvial plot illustrating how 55 assessment items transition across three dimensions: adherence level to item writing guidelines (High, Good, Fair), relevance to the target construct (Main Concept, Sub-Concept, Minor Concept, Inadequate), and cognitive demand based on Bloom’s Taxonomy (Analysis, Application, Comprehension, Knowledge).

Each block (strata) represents dimensions, and the flow is from adherence level → concept relevance → cognitive level. Flow width represents the number of items in that category combination.
