# Supplementary material for: Development and Validation of Pharmacology Concept Inventory for Concept‐Based Learning: Leveraging Theory, Expert Insights, and Student Perspectives
Source: Pharmacol Res Perspect. 2026 Mar 22;14(2):e70237. doi: 10.1002/prp2.70237 (PMC13140222; doi:10.1002/prp2.70237)
Supplement: Supplementary file 4 — Table S1: Pharmacology expert team composition for PCI tool item development and validation. [file PRP2-14-e70237-s004.docx]

**Table S1**: Pharmacology expert team composition for PCI tool item development and validation

| Expert Identified | Country | Highest Qualification | Teaching Experiences (in years) | Students enroll per cohort | Discipline primarily teaches | Students’ cohort Major Category | Student cohort in addition to major category | Additional course | Additional qualifications |
| --- | --- | --- | --- | --- | --- | --- | --- | --- | --- |
| Female | Australia | PhD | > 20 | 51-100 | Systems pharmacology/ therapeutics | Medical students | BSc, BMedSci | * | * |
| Female | Australia | PhD | > 20 | 101-150 | Systems pharmacology/ therapeutics | BMedSci students | * | * | * |
| Female | Australia | PhD | 6 - 10 | 151-200 | Basic pharmacology | BMedSci students | PhD students | MD, Chiropractic, Physiotherapy, Medicinal Chemistry | BPharm, MPH |
| Male | Australia | PhD | > 20 | >250 | Basic pharmacology | Pharmacy students | Pharmaceutical Science |  |  |
| Male | Australia | PhD | > 20 | 201-250 | Medicinal chemistry | Pharmacy students | Pharmaceutical Science (UG and PG) | * | BPharm, MPharm |
| Female | Australia | PhD | >20 | 100-250 | Basic pharmacology | BMedSci students | Science, Allied Health (Physiotherapy, Podiatry, Orthoptics), Paramedicine, Vet Science | * | GradCertHigherEd |
| Female | Australia | PhD | > 20 | >250 | Basic pharmacology | BMedSci students | Science, Pharmacy, Medicine, Dentistry, Nursing | Systems pharmacology/ therapeutics | BSc (Hons), Grad Dip Sc (Psych) |
| Female | Canada | PhD | 6 - 10 | >250 | Basic pharmacology | Medical students | Medicine; Dentistry; UG students (Science, medical, dental and nurse) | Clinical pharmacology; asynchronous course to nursing | B. of Pharmacy |
| Male | Hong Kong | PhD | > 20 | >250 | Basic pharmacology | Pharmacy students | BMedSci, Nursing, Medical, PG | * | PDip |
| Male | India | Master (MD) | 11 - 20 | 101-150 | Clinical Pharmacology | Medical students | PhD students, Nursing, BSc Optometry | * | PG Diploma (Diabetology) |
| Female | Ireland | PhD | 11 - 20 | 51-100 | Basic pharmacology | Science students | Medical students | Pharmacy | M.Ed. Higher Education Studies |
| Male | Ireland | PhD | > 20 | 201-250 | Basic pharmacology | Medical students | Science, Nursing, Podiatry, Postgraduate | * | MA Academic Practice |
| Female | Malta | PhD | > 20 | 101-150 | Pharmacokinetics | Medical students | Pharmacy; pharmacology | all health care professionals; psychologists | Pharmacist, PhD Pharmacology |
| Male | Sweden | PhD | 11 - 20 | 51-100 | Basic pharmacology | Pharmacy students | Dentistry, Nursing, Biomedical etc. | Medicine | Pharmacist, PhD |
| Female | The Netherlands | PhD | > 20 | >250 | Basic pharmacology | Pharmacy students | Biomedical Science, Life Sciences, and medical students | * | * |
| Female | UK | PhD | 11 - 20 | >250 | Basic pharmacology | Medical students | * | * | * |
| Male | UK | PhD | 11 - 20 | 51-100 | Systems pharmacology/ therapeutics | BMedSci students | BSc Pharmacology | * | * |
| Female | UK | PhD | > 20 | 101-150 | Basic pharmacology | Medical students | Medical Physiology and Therapeutics BSc | * | * |
| Female | UK | PhD | 11 - 20 | 101-150 | Basic pharmacology | Science students | BSc Pharmacology joint hons with Immunology/ Micro/Biochemistry; MSc Advanced Pharmacology | Biomedical Sciences | PGDip in Academic Practice |
| Male | UK | MSc | 6 - 10 | 151-200 | Systems pharmacology/therapeutics | Veterinary medicine students | Pharmacy, Nursing, Paramedicine, Allied Health (Midwifery, Physiotherapy) | Pharmacy  Independent/ Supplementary Prescribers | Bachelor’s in pharmacy, Veterinary Medicine, Natural Sciences; Masters in Medicinal Chemistry, Higher Education |
| Female | UK | PhD | 6 - 10 | 151-200 | Pharmacokinetics | BMedSci students | PGT Drug Discovery | Pharmacy | Bachelor’s pharmacy (BPharm), MSc Clinical Pharmacy |
| Male | UK | PhD | 11 - 20 | 51-100 | Basic pharmacology | Science students | Medical, Dental, PGT | Clinical pharmacology | * |
| Female | USA | PhD | > 20 | >250 | Clinical Pharmacology | Medical students | PA | PA | PharmD |
| Female | USA | PhD | 11 - 20 | 151-200 | Systems pharmacology/ therapeutics | Medical students | PhD students; PA; Anesthesiologist Assistant | * | * |
| Female | USA | PhD | > 20 | 51-100 | Systems pharmacology/ therapeutics | Medical students | * | * | Pharmacist (RPh) |
| Female | USA | PhD | 11 - 20 | 101-150 | Systems pharmacology/ therapeutics | Medical students | PhD, PA, Pharmacy, Dental | Dental, PhD | * |
| Male | USA | PhD | 11 - 20 | 51-100 | Basic pharmacology | Medical students | * | * | * |

*: Information not provided; BMedSci: Biomedical Science; BSc: Bachelor of Sciences; DSc: Doctor of Science; GI: Gastrointestinal; GradCertHigherEd: Graduate Certificate of Higher Education; GU: Genitourinary; MSc: Master of Sciences; PA: Physician Assistant; PD: Pharmacodynamics; PG: Postgraduate; PGT: Postgraduate Taught Course; PK: Pharmacokinetics; UG Undergraduate
